# Biased statistical ensembles for developable ribbons

**DOI:** 10.1073/pnas.2221419120

**Published:** 2023-10-04

**Authors:** G. H. M. van der Heijden, E. L. Starostin

**Affiliations:** ^a^Department of Civil, Environmental and Geomatic Engineering, University College London, London WC1E 6BT, United Kingdom; ^b^School of Engineering, London South Bank University, London SE1 0AA, United Kingdom

Yong et al. (1) use a numerical method (2) that has previously been shown to be flawed (3) and produce statistical results for developable ribbons that contradict results in the literature (4).

The problem with their numerical method is that it is based on the Frenet frame of tangent, principal normal and binormal of the discrete chains computed for submission to the ensemble averaging. The Frenet frame is well known to be an improper choice of frame for ribbon modeling (5): It has the property that it flips at inflection points of the ribbon centerline, i.e., the principal normal and binormal change sign, becoming opposite to the continuous vectors of a material frame. The frame therefore loses contact with the physical deformation of the ribbon. Yong et al. do not allow for these Frenet frame flips (ϕ→ϕ−π); instead, they assume highly twisted segments (with large azimuthal angle ϕ), which are then strongly penalised by the energy functional. They therefore ignore inflection points.

As explained in ref. (3), the problem can be cured by working with different ranges of angles. Taking advantage of the fact that for developable ribbons the Frenet and material frames are locked/coincident everywhere except at inflection points, one can effectively work with a material frame by defining −π≤θ<π, −π/2≤ϕ<π/2 instead of the choice 0≤θ<π, 0≤ϕ<2π taken in refs. (1) and (2). This eliminates jumps by π in the azimuthal angle $\phi_{i}$ (flips) between binormals $b_{i}$ and allows for inflections by means of a sign change of the polar angle $\theta_{i}$ between tangents $t_{i}$. Doing this, one finds exponential decay of the tangent–tangent correlation function $<t_{n}\cdot t_{0}>$ (3), also found in ref. 4 for developable ribbons, in contrast to the oscillatory decay predicted by Yong et al.

**Fig. 1.**
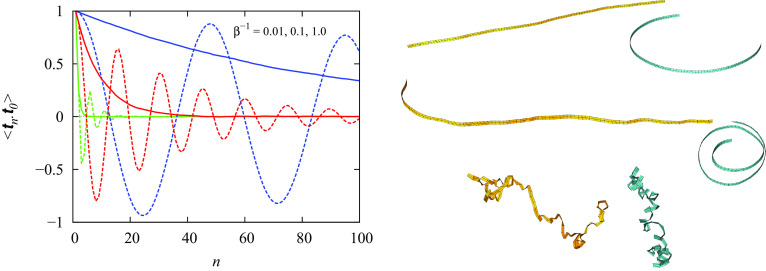
Results of the Monte Carlo simulation. (*Left*) Tangent–tangent correlation function $<t_{n}\cdot t_{0}>$ for 1/β=0.01 (blue), 0.1 (red), and 1.0 (green) for both Yong et al.’s angles (dashed) and our proposed corrections (solid). (*Right*) Typical equilibrated ribbon shapes (blue for Yong et al.’s angles, yellow for corrected angles, for the same 1/β values, increasing down), drawn with fictitious width. Pairs of solutions are equally scaled, but the scaling varies between pairs.

Here, we further strengthen our claim by performing Monte Carlo simulations to compare results for the two different choices of angles at finite temperature. We take the same parameters as in ref. 1, i.e., n=100, $10^{6}$ sweeps (but $10^{7}$ for 1/β=0.01), half of which are used for equilibration. $1/\beta =ak_{B}T/\left(Bw\right)$ is the dimensionless temperature. No end loads are applied. Results are displayed in Fig. 1.

Fig. 1, *Left* confirms oscillatory decay of $<t_{n}\cdot t_{0}>$ for the angles in ref. 1 and exponential decay for our angles. The results for 1/β=0.01 (solid blue curve) give a normalised persistence length $l_{p}/a=91.12$, consistent with the exact asymptotic limit (32/35)β=91.42 (as 1/β→0) derived in ref. 3. Binormal–binormal correlation functions (not shown) are identical for the two choices of angles.

Fig. 1, *Right* shows typical equilibrated ribbon shapes for both types of angles. The (unphysical) blue ribbons appear to collapse into a tight coil as temperature decreases but then uncoil and approach the straight configuration in the zero-temperature limit. The blue solutions have no inflections, while the yellow solutions on average have inflections at 50 (out of 100) segments, corresponding to sign changes of the enclosed $\theta_{i}$. The elastic energy of the ribbon is in both cases identically distributed, with, for 1/β=0.1, average Ea/(Bw)=15 and standard deviation 1.3.

Yong et al.’s different results reflect the artificial helical bias built into their statistical ensemble by rejecting inflected-ribbon configurations. This bias is not particular to Sadowsky ribbons: Use of the Frenet frame similarly causes statistical bias in other ribbon/rod models (e.g., (6)) in which torsion is penalised, i.e., in which the energy depends on ϕ.
